# Severe Exercise and Exercise Training Exert Opposite Effects on Human Neutrophil Apoptosis via Altering the Redox Status

**DOI:** 10.1371/journal.pone.0024385

**Published:** 2011-09-09

**Authors:** Guan-Da Syu, Hsiun-ing Chen, Chauying J. Jen

**Affiliations:** 1 Institute of Basic Medical Sciences, National Cheng Kung University Medical College, Tainan, Taiwan; 2 Department of Physiology, National Cheng Kung University Medical College, Tainan, Taiwan; Mayo Clinic, United States of America

## Abstract

Neutrophil spontaneous apoptosis, a process crucial for immune regulation, is mainly controlled by alterations in reactive oxygen species (ROS) and mitochondria integrity. Exercise has been proposed to be a physiological way to modulate immunity; while acute severe exercise (ASE) usually impedes immunity, chronic moderate exercise (CME) improves it. This study aimed to investigate whether and how ASE and CME oppositely regulate human neutrophil apoptosis. Thirteen sedentary young males underwent an initial ASE and were subsequently divided into exercise and control groups. The exercise group (n = 8) underwent 2 months of CME followed by 2 months of detraining. Additional ASE paradigms were performed at the end of each month. Neutrophils were isolated from blood specimens drawn at rest and immediately after each ASE for assaying neutrophil spontaneous apoptosis (annexin-V binding on the outer surface) along with redox-related parameters and mitochondria-related parameters. Our results showed that i) the initial ASE immediately increased the oxidative stress (cytosolic ROS and glutathione oxidation), and sequentially accelerated the reduction of mitochondrial membrane potential, the surface binding of annexin-V, and the generation of mitochondrial ROS; ii) CME upregulated glutathione level, retarded spontaneous apoptosis and delayed mitochondria deterioration; iii) most effects of CME were unchanged after detraining; and iv) CME blocked ASE effects and this capability remained intact even after detraining. Furthermore, the ASE effects on neutrophil spontaneous apoptosis were mimicked by adding exogenous H_2_O_2_, but not by suppressing mitochondrial membrane potential. In conclusion, while ASE induced an oxidative state and resulted in acceleration of human neutrophil apoptosis, CME delayed neutrophil apoptosis by maintaining a reduced state for long periods of time even after detraining.

## Introduction

Neutrophils play a critical role in the first line of defense against pathogens. They rapidly migrate to the infection site, ingest the pathogens, release reactive oxygen species (ROS) to kill the pathogens, and even release their own DNA to form extracellular traps [1]. Regulation of neutrophil life span by spontaneous apoptosis has for a long time been considered to be a major way of optimizing innate immunity under healthy conditions. Accelerated neutrophil apoptosis often results in neutropenia and can be associated with bacterial and fungal infections [2]. In contrast, inhibition of apoptosis prolongs neutrophil survival and contributes to the accumulation of these cells at inflammatory sites [3]. However, neutrophil spontaneous apoptosis highly depends on ROS level and mitochondria integrity [4]. There are three lines of evidence supporting that ROS limits the neutrophil lifespan. First, exogenously added oxidant accelerates neutrophil apoptosis [5] and antioxidant delays it [6], [7]. Second, neutrophils from patients with impaired NADPH oxidase (an enzyme complex which produces ROS) increase neutrophil lifespan as compared with neutrophils from healthy subjects [8], [9]. Third, neutrophil glutathione (GSH, a major antioxidant in mamalian cells) decreases over time, results in ROS accumulation, and limits neutrophil lifespan [10]. Although mitochondrial is generally considered a major source of ROS, whether mitochondrial ROS (mtROS) participate in neutrophil apoptosis is unclear. Neutrophil mitochondria hardly participate in energy production but usually involve in apoptosis regulation instead [11]. Apoptotic neutrophils are functionally compromised because their stimulation-evoked responses, such as spreading, chemotaxis and oxidative burst, are either completely absent or greatly reduced [12].

Despite the crucial role of neutrophils in innate immunity, how neutrophil spontaneous apoptosis is regulated under physiological conditions in healthy subjects is not fully understood. Exercise is believed to be a natural way to modulate immunity; it can be generally divided into acute severe exercise (ASE) and chronic moderate exercise (CME), according to exercise intensity, duration and frequency. ASE increases tissue damage and oxidative stress [13], [14], and stimulates the secretion of many pro-inflammatory cytokines, such as TNF-α, IL-6, and IL-1β [15]–[17]. It also increases the risk of upper respiratory tract infection [18], [19]. In neutrophils, some reports indicate that ASE paradigms directly stimulate the neutrophil ROS release [20]–[22], while other studies using well-trained subjects show otherwise [23]–[25]. These controversies could be due to differences in the physical fitness of subjects, exercise protocols, and the assay methods for neutrophil ROS [26], [27]. Recent human studies show that repeated ASE increases apoptosis and reduces mitochondrial membrane potential (ΔΨm) in neutrophils [28], [29]. Until now, the roles of ROS and mitochondria in ASE-evoked neutrophil apoptosis are unclear. In contrast to ASE, few studies have investigated how CME affects neutrophil apoptosis [30]. Nevertheless, CME lowers the levels of many pro-inflammatory cytokines (e.g., C-reactive protein, IL-1, and IL-6) and elevates the level of IL-10, an anti-inflammatory cytokine in the circulation [31]. CME improves immunity in general as indicated by lowered susceptibility to viral and bacterial infections [18], [19], [32].

Since ASE and CME have opposite effects on inflammatory cytokines, they may differentially regulate neutrophil apoptosis as well. This new concept has been supported by our recent study, that is, neutrophil apoptosis is delayed by 2-month CME [30]. However, it is not clear whether ROS and mitochondria are involved in the apoptosis delaying effect. Moreover, how ASE and CME exert opposing effects on neutrophil apoptosis have not been addressed before. Therefore, we hypothesize that alterations in redox status and mitochondria integrity are responsible for the opposing effects of ASE and CME on human neutrophils apoptosis. We further propose that CME is able to prevent the ASE-evoked neutrophil apoptosis possibly by maintaining redox status and mitochondria integrity. To address these issues, sedentary healthy males underwent an initial ASE and were subsequently divided into exercise and control groups. The exercise group underwent 2 months of CME followed by 2 months of detraining (DT). Additional ASE paradigms were performed at the end of each month. Neutrophils were isolated from blood specimens drawn at rest and immediately after each ASE for assaying neutrophil spontaneous apoptosis along with redox-related and mitochondria-related parameters.

## Materials and Methods

### Ethics statement

The protocol was reviewed according to the Declaration of Helsinki and approved by the Human Ethics Committee of National Cheng Kung University Medical College (IRB #: ER-96-92). Written informed consent was received from all participants.

### Subjects

The exercise paradigms (ASE, CME and DT) were modified from our previous reports [33], [34]. Briefly, 13 healthy sedentary male volunteers aged between 20 and 24 years participated in this study. They fulfilled the following requirements: no regular exercise (≤1 time per week) in the past 6 months, no smoking, no previous medical record of cardiovascular or metabolic diseases, no recent symptoms of upper respiratory tract infection, and abstained from any medication for at least 1 month before the study. All subjects were involved in studying the effects of initial ASE. They were then randomly divided into exercise group (8 subjects) and sedentary control group (5 subjects). There were no significant differences between groups in the initial anthropometric data and exercise performance data (Table 1).

**Table 1:** Basic physiological parameters and exercise performance in exercise and sedentary groups.

	Exercise group (22±0 year old)					Sedentary group (22±1 year old)		
	Initial	1^st^ month	2^nd^ month	3^rd^ month	4^th^ month	Initial	2^nd^ month	4^th^ month
Body weight (kg)	64±2	62±2*	62±2*	63±2	63±2	66±3	65±3	66±3
Body mass index (kg/m^2^)	21.7±0.6	21.2±0.5*	21.1±0.6*	21.5±0.6	21.5±0.6	22.6±0.8	22.3±0.9	22.6±0.9
Resting heart rate (bpm)	70±3	64±3	63±2*	71±5	69±3	65±2	67±1	66±3
ASE duration (min)	31±2	40±1*	48±2*	44±1*	42±2*	30±2	30±3	29±2
Maximum workload (watt)	109±7	135±4*	165±7*	152±5*	140±5*	104±6	105±10	100±7
Maximum heart rate (bpm)	184±5	184±3	185±2	186±3	186±3	184±2	182±3	182±3

Data were analyzed by one-way ANOVA with repeated measures followed by Bonferroni post-test.

*p<0.05, compared with initial values. The differences between exercise (n = 8) and sedentary control (n = 5) groups were insignificant at the beginning as analyzed by unpaired *t*-tests. There was no time-dependent effect in the sedentary control group.

### Exercise paradigms and blood collection

Subjects in both groups arrived at 09:00 and rested for about 30 min. All subjects performed the initial ASE on a cycle ergometer with continuous increments of work load every 3 min until exhaustion. The heart rate reached at least 90% of the predicted maximal heart rate (198 bpm) at the end of ASE. Subjects in the exercise group undertook 2 months of CME (30 min a day, 5 days a week at 60% of maximal workload determined by the initial ASE and adjusted 1 month later) followed by 2 months of DT (abstained from regular exercise of any form). Throughout the experimental period, subjects in the exercise group underwent ASE tests once a month. Sedentary control subjects maintained their sedentary life-style for 4 months, and received ASE tests once every 2 months. Peripheral venous blood samples were drawn at rest and immediately after each ASE. Blood specimens were anti-coagulated with sodium citrate and stored on ice. Neutrophils collected before each ASE were defined as “resting” specimens.

### Neutrophil isolation and culture

Neutrophils purified by density gradient centrifugation were washed in Hanks' balance buffer and shocked in a 0.2% NaCl hypotonic solution for 30 sec to remove contaminating erythrocytes. Part of the specimen was frozen for later measurement the GSH and glutathione disulfide (GSSG) level. The remaining was resuspended at 5×10^6^ cells/ml in RPMI 1640 medium supplemented with 10% fetal calf serum. These freshly isolated neutrophils were either used immediately for measuring redox-related parameters or cultured for various time periods (at 37°C in the presence of 5% CO_2_) for measuring apoptosis-related parameters. The neutrophil purity and viability (>95%) were routinely checked by Wright's stain and trypan blue exclusion, respectively.

### Measurement of neutrophil redox status

Neutrophil cytosolic ROS, in the presence or absence of phorbol myristate acetate (PMA), was estimated by using a fluorescent indicator (Fig. S1) [35]. Briefly, freshly isolated neutrophils were incubated with DCF-DA (1 µM; Sigma-Aldrich, St. Louis, MO, USA) for 20 min. DCF-DA fluorescence intensity was then recorded by flow cytometry before and after adding PMA (10 ng/ml, 10 min) to quantify the levels of basal cytosolic ROS and PMA-stimulated cytosolic ROS. Total GSH and GSSG in neutrophils were assayed by GSH reductase recycling methods [36]. After removing proteins by 5% sulfosalicylic acid, samples were mixed with DTNB and GSH reductase (Sigma-Aldrich) for 2 min. After adding NADPH the colorimetric reaction kinetics was measured to calculate the total GSH level in nmole/mg protein. For GSSG assay, deproteinized samples were treated with 2-vinylpyridine (Sigma-Aldrich) for 1 h to remove existing GSH and this reaction was terminated by adding triethanolamine (Sigma-Aldrich). The GSSG level was then analyzed by the same GSH reductase recycling methods. The reduced GSH level was calculated as the total GSH – 2×GSSG. Finally, the intracellular oxidation level was indexed by the ratio between GSSG and the reduced GSH, i.e., GSSG/GSH.

### Measurements of neutrophil apoptosis and mitochondria-related parameters

Neutrophil apoptosis and mitochondria-related parameters included Annexin-V (Ann-V) binding, ΔΨm, and mtROS (Fig. S1). Ann-V binding on the outer surface was used as an apoptosis marker. Neutrophil ΔΨm was quantified by measuring the red fluorescence intensity of JC-1 [37]. MitoSOX was used to detect mtROS [38]. Freshly isolated neutrophils were incubated for 0, 4 and 10 h in culture and then stained with JC-1 (7.7 µM; Invitrogen, Carlsbad, CA, USA), Ann-V (×20 dilution; Invitrogen), MitoSOX (5 µM; Invitrogen), or MitoTrakcer Red (250 nM; Invitrogen) at 37°C for 20 min. The fluorescence intensity (FI) was analyzed by flow cytometry.

### Elevation of neutrophil ROS *in vitro*

To clarify the role of ROS in effects of ASE on neutrophils, 9 additional sedentary subjects (age 21±1 yr, body weight 67±3 kg, height 175±3 cm, resting heart rate 68±2 bpm) were recruited for neutrophil ROS manipulation experiments. To mimic the oxidative stress during the initial ASE, neutrophils isolated at rest were incubated with freshly prepared H_2_O_2_ (0, 100 or 1000 µM) for 30 min. After H_2_O_2_ exposure, these neutrophils were washed and resuspended in the medium for various time periods (0, 4 or 10 h). Parameters related to neutrophil apoptosis and redox status were performed as described before.

### Reduction of neutrophil ΔΨm *in vitro*

To verify the role of neutrophil ΔΨm reduction in neutrophil apoptosis and ROS generation, neutrophils isolated from 6 additional sedentary subjects (age 21±1 yr, body weight 67±2 kg, height 173±1 cm, resting heart rate 72±5 bpm) were recruited for neutrophil ΔΨm manipulation experiments. Resting neutrophils were incubated with various concentrations (0, 10 and 100 nM) of carbonyl cyanide-p-trifluoromethoxyphenylhydrazone (FCCP; Sigma-Aldrich) in the Hank's balance buffer for various time periods (0, 4 or 10 h) to reduce neutrophil ΔΨm [39]. Parameters related to neutrophil apoptosis and ROS level were performed as described before.

### Statistical analysis

Unpaired *t*-test was used to analyze the differences between exercise and sedentary control groups. Data from the same subjects were analyzed by various paired or repeated analysis. Paired *t*-test was used to analyze the effects of ASE, H_2_O_2_, or FCCP before and after treatments in the self-controlled studies. One-way ANOVA with repeated measures followed by Bonferroni post-test was used to analyze various time-dependent or dose-dependent effects of single treatments. Two-way ANOVA with repeated measures followed by Bonferroni post-test was used to analyze interactions between initial ASE effects and apoptosis kinetics, between CME effects and apoptosis kinetics, and between ASE effects and CME-DT states. Significant differences were defined as *p*<0.05. All data were presented as mean ± SEM, where n was the number of subjects.

## Results

### Exercise effects on basic physiological parameters and exercise performance

This study lasted 4 months and used a combination of various exercise paradigms, i.e., ASE once every month, CME for 2 months, and followed by another 2 months of DT. Results in Table 1 show the time-dependent effects of exercise on basic physiological parameters and exercise performance. Two-month CME reduced the basic physiological parameters (body weight, body mass index, and resting heart rate) and increased both exercise time and maximal workload. The CME effects on physiological parameters were reverted to the pre-training state after 1 months of DT, while effects on exercise performance were partially reversed after 2 months of DT. All parameters remained unchanged in the sedentary control group.

### Effects of ASE on neutrophil apoptosis

**Figure 1:**
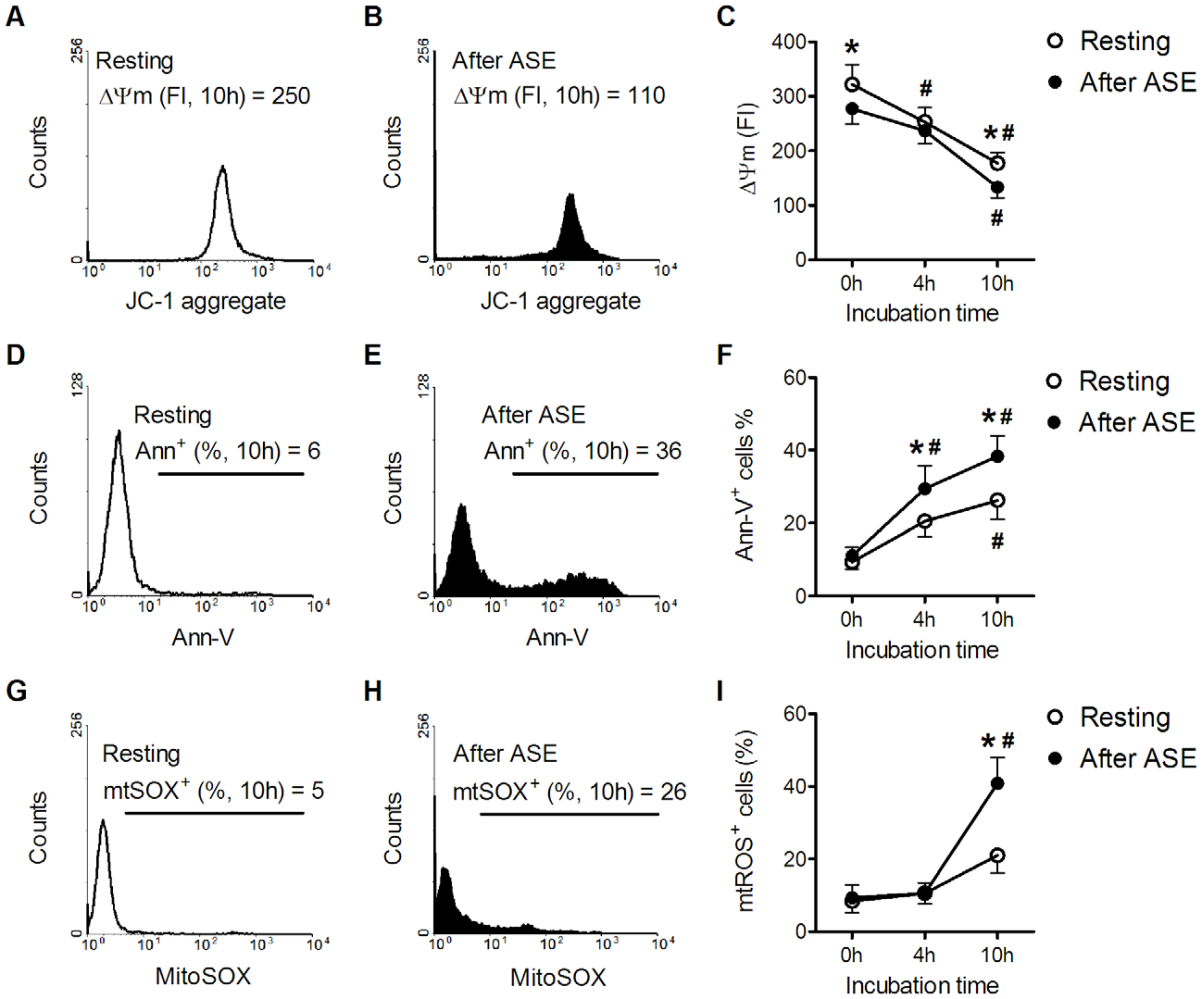
Effects of ASE on neutrophil apoptosis. Blood specimens were obtained from all subjects at rest and immediately after ASE. Apoptosis assays were carried out using neutrophils cultured *in vitro* for 0, 4 and 10 h. The neutrophil ΔΨm was quantified by JC-1 aggregate FI (A–C), Ann-V binding by the fraction of Ann-V^+^ cells (D–F), and mtROS by the fraction of MitoSOX^+^ cells (G–I). Data in (C, F, I) were analyzed by two-way ANOVA with repeated measures followed by Bonferroni post-test. * *p*<0.05, after ASE *vs* resting; # *p*<0.05 compared with corresponding specimens at 0 h; n = 13.

Neutrophils from all subjects (n = 13) were isolated at rest and immediately after the initial ASE. Cells were subsequently cultured *in vitro* for up to 10 h to determine the kinetics of apoptosis (Ann-V binding) and mitochondria-related parameters (ΔΨm and mtROS) (Fig. 1). Since apoptotic neutrophils showed gradual changes of Ann-V binding, ΔΨm, and mtROS, these three parameters are considered as “apoptosis-related parameters” in this study. The initial ASE accelerated all three apoptosis-related parameters sequentially: the ΔΨm reduction occurred immediate (0 h), followed by the elevation of apoptosis (4 h), and finally the elevation of mtROS (10 h). Taken together, ASE accelerated the process of neutrophil spontaneous apoptosis, leading by the ΔΨm suppression.

### Effects of ASE on neutrophil redox status

**Figure 2:**
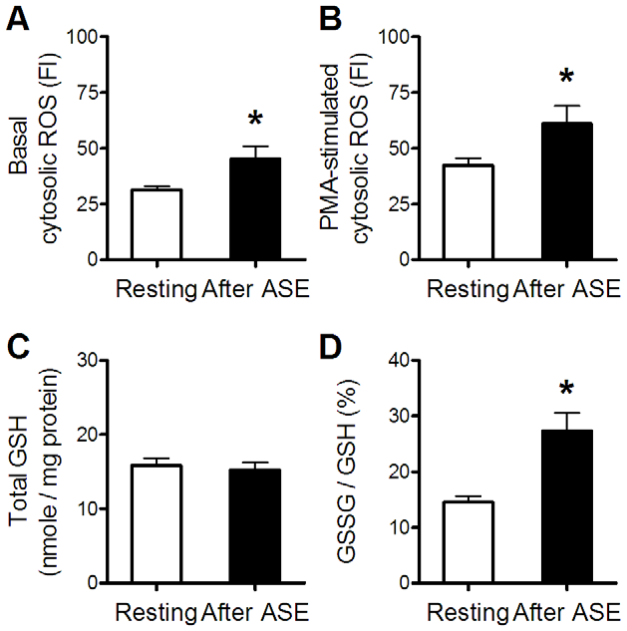
Effects of ASE on neutrophil redox status. Redox-related parameters of freshly isolated neutrophils at rest were compared with those immediately after the ASE. Neutrophil basal cytosolic ROS and PMA-stimulated cytosolic ROS were measured by DCF-DA fluorescence intensities before and after 10-min PMA stimulation, respectively (A, B). Neutrophil intracellular redox capacity was indicated by the total GSH amount (C). Neutrophil intracellular oxidation level was indicated by the GSSG/GSH ratio (D). Data were analyzed by paired *t*-test. * *p*<0.05, after ASE *vs* resting; n = 13.

Neutrophils freshly isolated at rest and immediately after the initial ASE were analyzed for intracellular redox status. The initial ASE significantly augmented the basal cytosolic ROS, the PMA-stimulated cytosolic ROS, and the GSSG/GSH ratio (Fig. 2). However, it altered neither the total GSH level (Fig. 2C) nor the oxidative burst (PMA-stimulated cytosolic ROS/basal cytosolic ROS; 1.37±0.11 *vs* 1.36±0.08, post-ASE *vs* pre-ASE, n = 13). Taken together, ASE shifted the neutrophil redox status toward a relatively oxidative state.

### Effects of CME and DT on redox status in resting neutrophils

**Figure 3:**
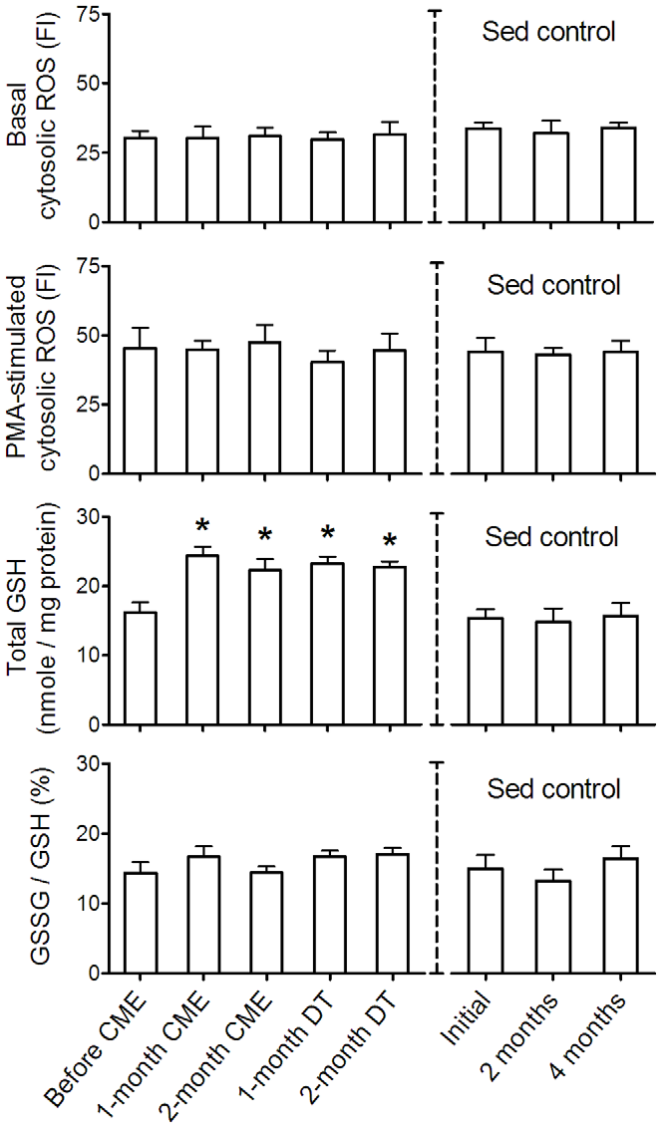
Effects of CME and DT on redox status in resting neutrophils. At various time points of the CME-DT paradigm, neutrophils were freshly isolated from subjects at rest to determine the redox-related parameters, i.e. basal cytosolic ROS, PMA-stimulated cytosolic ROS, total GSH, and GSSG/GSH ratio. Data were analyzed by one-way ANOVA with repeated measures followed by Bonferroni post-test. * *p*<0.05, compared with initial values. No differences between exercise (n = 8) and sedentary control (n = 5) groups were found at the beginning (analyzed by unpaired *t*-tests). There was no time-dependent effect in the sedentary control group.

At various time points of the CME-DT paradigm, neutrophils were collected at rest for the measurements of redox-related parameters. Unlike initial ASE, neither CME nor DT significantly affected the basal cytosolic ROS, the PMA-stimulated cytosolic ROS, and the GSSG/GSH ratio (Fig. 3). In contrast, the total GSH level was enhanced after 1 month of CME and remained high after 2 months of DT. All redox-related parameters remained unchanged in sedentary control group. Comparing with the pro-oxidative effects of ASE, the overall CME effects enduringly increased the antioxidant reserve without altering the basal redox status.

### Effects of CME and DT on apoptosis in resting neutrophils

**Figure 4:**
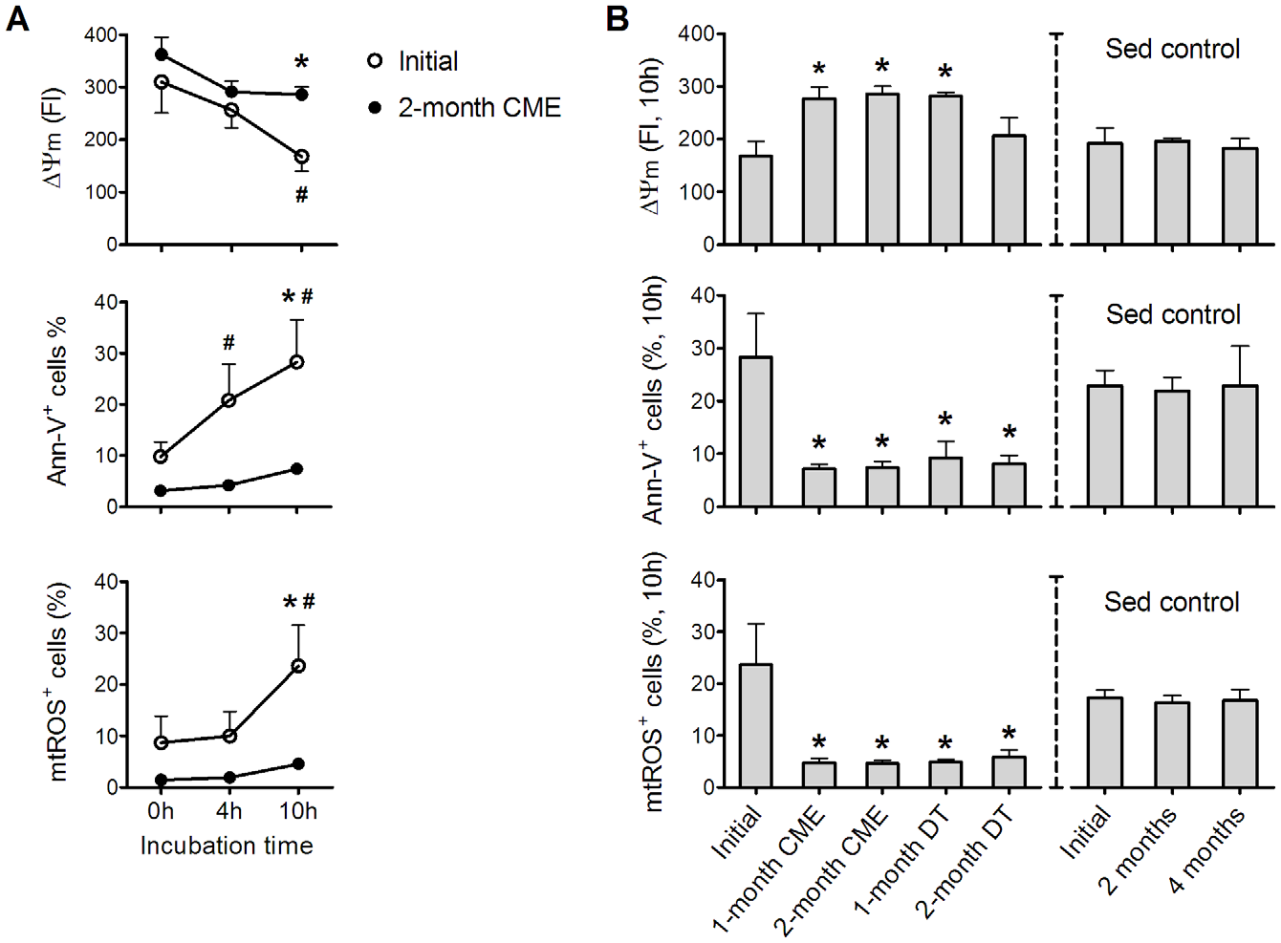
Effects of CME and DT on apoptosis in resting neutrophils. At various time points of the CME-DT paradigm, neutrophils isolated under resting conditions were subsequently cultured *in vitro* for up to 10 h to determine the apoptosis-related parameters (ΔΨm, Ann-V binding, and mtROS). (A): The kinetics of neutrophil apoptosis-related parameters before and after 2-month CME. (B): The CME and DT effects on apoptosis-related parameters measured after 10 h incubation *in vitro*. Data in (A) were analyzed by two-way ANOVA with repeated measures followed by Bonferroni post-test. Data in (B) were analyzed by one-way ANOVA with repeated measures followed by Bonferroni post-test. * *p*<0.05, compared with initial values; # *p*<0.05 compared with corresponding specimens at 0 h; n = 8. No differences between exercise (n = 8) and sedentary control (n = 5) groups were found at the beginning (analyzed by unpaired *t*-tests). There was no time-dependent effect in the sedentary control group.

At various time points of the CME-DT paradigm, neutrophils isolated under resting conditions were subsequently cultured *in vitro* for up to 10 h to determine the apoptosis-related parameters. Two-month of CME significantly delayed the progression of apoptosis-related parameters (Fig. 4A). Interestingly, the anti-apoptotic effects (including higher ΔΨm, lower Ann-V binding and lower mtROS) were observed as early as 1 month of CME and they remained effective even after 1 or 2 months of DT (Fig. 4B). Taken together, opposite to the pro-apoptotic effects of ASE, the effects of CME were anti-apoptotic and relatively long-lasting. Note: all of these apoptosis-related parameters in the sedentary control group remained the same in the experimental period.

### ASE effects were prevented by CME and partially restored by DT

In our hands, many CME effects and ASE effects on neutrophils were in the opposite direction. To investigate whether CME-DT could counteract the adverse effects ASE or not, the ASE effects (after ASE values / resting values) before and after CME-DT were determined and summarized in Table 2. Our results showed that initial ASE effects vanished after 1 month of CME and mostly remained vanished even after 2 months of DT. Moreover, the mitochondria obtained at different stages of CME-DT were functionally superior to those obtained at the beginning of exercise experiments, i.e., they showed higher ΔΨm along with lower mtROS both at rest and after ASE. Please note that the resting values in table 2 were adopted from those presented in Figs. 3 and 4B.

**Table 2:** ASE effects on neutrophils were prevented by CME and partially restored by DT.

After ASE/Resting	Initial	1-month CME	2-month CME	1-month DT	2-month DT
*Neutrophil redox-related parameters*					
Basal cytosolic ROS (FI)	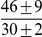 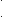 *	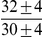	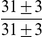	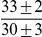	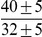
PMA-stimulated cytosolic ROS (FI)	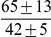 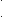 *	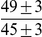	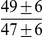	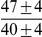	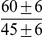 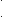 *
Total GSH (nmole/mg protein)	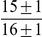	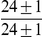	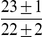	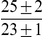	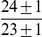
GSSG/GSH (%)	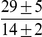 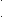 *	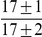	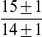	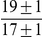	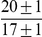
*Neutrophil apoptosis-related parameters*					
ΔΨm (FI, 10 h)	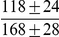 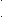 *	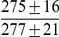	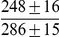	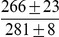	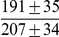
Ann-VP^+P^ cells (%, 10 h)	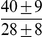 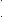 *	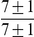	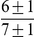	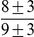	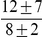
mtROSP^+P^ cells (%, 10 h)	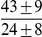 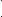 *	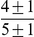	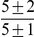	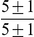	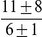

The ASE effects (After ASE *vs* resting) during CME and DT periods were analyzed by two-way ANOVA with repeated measures followed by Bonferroni post-test. The resting data were the same as presented in Figs. 3 and 4.

*p<0.05, after ASE *vs* resting, n = 8.

### Effects of exogenous H_2_O_2_ application on resting neutrophil redox status and apoptosis

**Figure 5:**
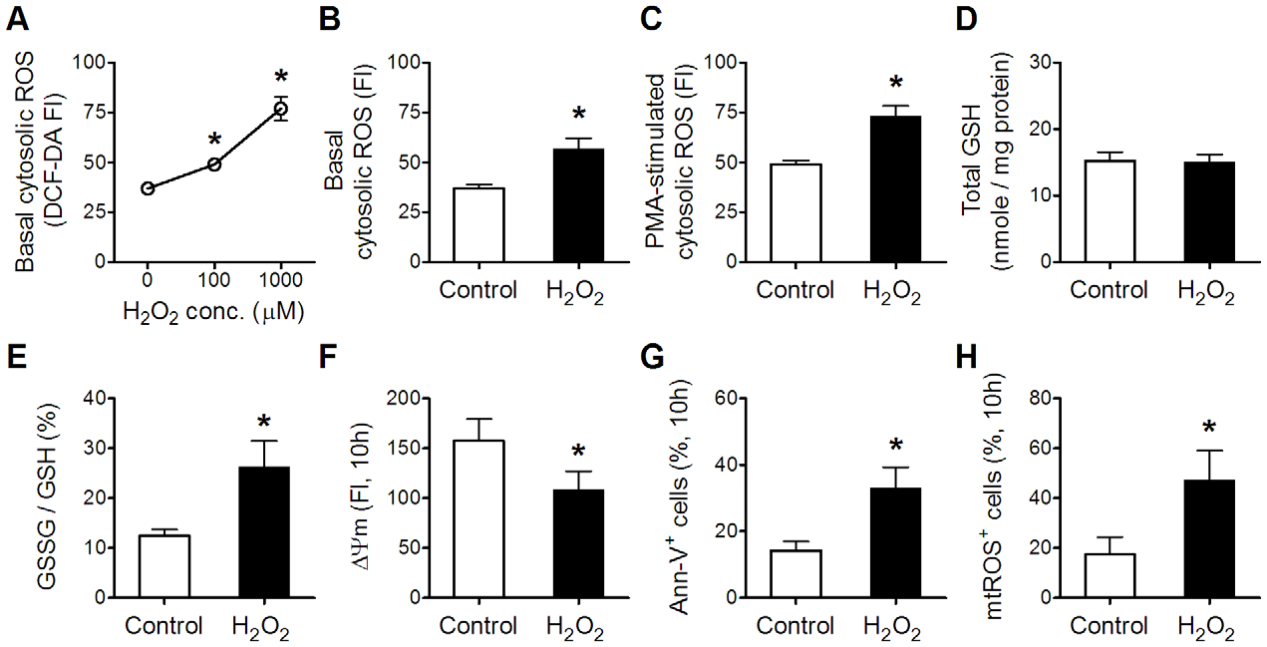
Effects of exogenous H_2_O_2_ application on resting neutrophil redox status and apoptosis. After 30-min H_2_O_2_ exposure, redox-related parameters of neutrophils were analyzed immediately (A–E), whereas the apoptosis-related parameters were determined in neutrophils cultured for 10 h (F–H). (A): neutrophil basal cytosolic ROS levels in response to different concentrations of H_2_O_2_ (0, 100, and 1000 µM). (B–E): effects of 100 µM H_2_O_2_ exposure on neutrophil basal cytosolic ROS, PMA-stimulated cytosolic ROS, total GSH, and GSSG/GSH. (F–H): effects of 100 µM H_2_O_2_ exposure on ΔΨm, Ann-V binding, and mtROS. Data in (A) were analyzed by one-way ANOVA with repeated measures followed by Bonferroni post-test. Data in other panels were analyzed by paired *t*-test. * *p*<0.05, H_2_O_2_ exposure *vs* untreated control; n = 9.

To test the hypothesis that redox status changes were the underlying mechanism responsible for the neutrophil apoptosis facilitated by a single bout of ASE, resting neutrophils were exposed to H_2_O_2_ for 30 min. We selected 100 µM H_2_O_2_ because it mimicked the initial ASE effects, i.e., both induced similar changes in ΔΨm and Ann-V binding (Figs. S2 and S3). As in post-ASE neutrophils (Figs. 1 and 2), these H_2_O_2_-treated neutrophils underwent comparable changes of redox- and apoptosis-related parameters (Fig. 5). Like ASE, H_2_O_2_ exposure did not alter the oxidative burst (PMA-stimulated cytosolic ROS/basal cytosolic ROS; 1.53±0.16 *vs* 1.59±0.21, H_2_O_2_ *vs* control, n = 8).

### Effects of ΔΨm reduction on ROS levels and apoptosis in resting neutrophils

**Figure 6:**
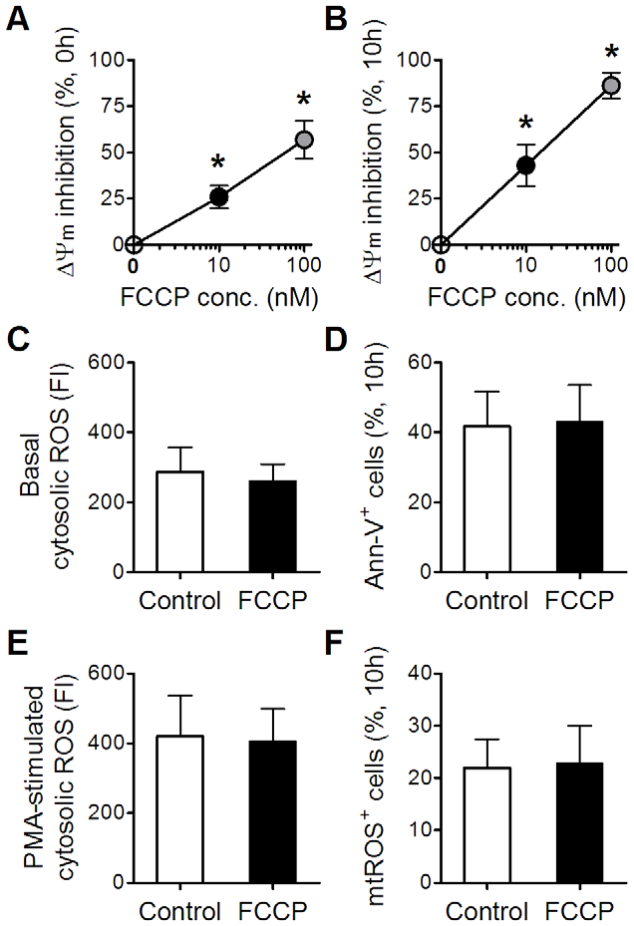
Effects of ΔΨm reduction on ROS levels and apoptosis in resting neutrophils. Freshly isolated neutrophils were incubated with FCCP (0, 10, and 100 nM) for 20 min (A) or 10 h (B) before ΔΨm determination. Neutrophils were incubated with 10 nM of FCCP for 20 min before measuring basal cytosolic ROS and PMA-stimulated cytosolic ROS (C, E). Neutrophils were incubated with 10 nM of FCCP for 10 h before measuring Ann-V binding and mtROS (D, F). Data in (A, B) were analyzed by one-way ANOVA with repeated measures followed by Bonferroni post-test. Data in (C–F) were analyzed by paired *t*-test. * *p*<0.05, FCCP *vs* untreated control; n = 6.

Because ΔΨm reduction was the early event after ASE, whether this alteration led to changes of neutrophil ROS level and apoptosis was studied by using FCCP to block ΔΨm. We selected 10 nM FCCP because it induced ΔΨm reductions compatible to those after ASE (Fig. 6). However, FCCP did not significantly affect basal cytosolic ROS, PMA-stimulated cytosolic ROS, Ann-V binding, and mtROS. In our hands, even 1000 nM of FCCP (enough to reduce about 80% ΔΨm in freshly isolated neutrophils) was unable to alter either ROS-related or apoptosis-related parameters (data not shown).

## Discussion

This study is the first to show that ASE and CME differentially affect human neutrophil apoptosis and redox balance. ASE accelerated neutrophil apoptosis, while CME damped it. The ASE-accelerated neutrophil apoptosis was likely to be mediated by elevated ROS, but not by depressed mitochondria membrane potential. Moreover, the ASE effects were diminished by CME due to increased neutrophil GSH to prevent ROS elevation. Finally the CME effects were relatively long-lasting, remained largely intact after ceasing regular exercise for 2 months.

Our results showed that a single bout of ASE in sedentary subjects was sufficient to increase redox- and apoptosis-related parameters in a sequential manner, i.e., cytosolic ROS elevation, GSH oxidation and ΔΨm reduction at 0 h, Ann-V binding increase at 4 h, and mtROS elevation at 10 h. Since both cytosolic ROS level and ΔΨm were altered immediately after ASE, their relative roles in modulating neutrophil apoptosis deserved detailed evaluation. Although ROS elevation and ΔΨm reduction cause damages in many cell types, their reaction mechanisms are subtly different in neutrophils [4]. The spontaneous apoptosis of neutrophils is initiated by ROS accumulation along with GSH decomposition, followed by death receptor activation in the plasma membrane [10]. The downstream of death receptor signaling then leads to ΔΨm reduction and mitochondrial disintegration. Consistently, our results showed that both ASE and exogenous H_2_O_2_ shifted the redox status towards oxidation and therefore inducing neutrophil apoptosis. Besides GSH, other antioxidants (GSH peroxidase and GSH reductase) are also decreased after ASE [21], [40], [41]. In contrast, mtROS apparently was not involved in the initial stage of ASE-accelerated neutrophil apoptosis because it did not rise until after 10 h incubation. Since, ROS activates neutrophil NF-κB and induces many pro-inflammatory cytokines [42], [43], the ASE-evoked oxidative stress would shift neutrophils toward pro-inflammatory and pro-apoptotic states, thus explaining at least in part the adverse effects of ASE on the innate immunity.

As a comparison, ΔΨm reduction is a sign of apoptosis in many cell types, since mitochondria dysfunction hampers ATP generation and affects almost all ongoing biochemical processes. However, our ΔΨm reduction experiments indicated that other neutrophil apoptosis-related parameters were unaffected by FCCP treatment. Perhaps the FCCP treatment alone *in vitro* was insufficient to cause further destruction of mitochondria, such as the release pro-apoptotic proteins. Neutrophils have relatively few mitochondria and their ATP generation is highly dependent on glycolysis, not ΔΨm [11], [44]. Although ΔΨm reduction did not accelerate neutrophil spontaneous apoptosis, it could impair chemotaxis, one of the major functional parameters in neutrophils [39].

Opposite to the adverse effects of ASE, the effects of CME were anti-oxidative and anti-apoptotic. CME elevated the total GSH level without altering the basal redox state in freshly isolated resting neutrophils. Since ROS accumulation initiates spontaneous apoptosis [10], the CME-induced GSH elevation conceivably delayed the progression of spontaneous apoptosis via retarding ROS accumulation. ROS reduction blocks neutrophil NF-κB activation and thus reduces the release of pro-inflammatory cytokines [45]. Interestingly, our recent report shows that CME retards neutrophil apoptosis by upregulating the iNOS-NO-cGMP-Mcl-1 pathway, indicating that small amounts of NO serves as a signaling molecule instead of a form of ROS [30]. Therefore, the beneficial effects of CME on immunity may, at least in part, be due to the enhanced anti-oxidative and anti-apoptotic effects on neutrophils.

It is conceivable that ASE augments neutrophil cytosolic ROS level via the ASE-evoked ROS elevation in the plasma and consequently accelerates neutrophil apoptosis. ROS released from skeletal muscles under ASE transiently tips the redox balance in the blood stream toward pro-oxidative state [46], [47], which could directly or indirectly induce cytosolic ROS elevation in neutrophils. As ROS can damage macromolecules essential for cellular functions, ROS could regulate neutrophil apoptosis by altering certain pro-apoptosis or anti-apoptosis molecules. Several potential ROS targets have been reported in neutrophils. ROS increases neutrophil ceramide generation, death receptor clustering, and thus accelerates apoptosis [10]. ROS also facilitate the release of cathepsin D from granules and consequently increases the cytosolic activity of caspase-8 [48]. Finally, ROS might influence the degradation of anti-apoptotic proteins crucial for neutrophil survival, such as Mcl-1 [49] and PCNA [50], [51]. Therefore, modulations of neutrophil redox status by exercise inevitably influence neutrophil apoptosis.

CME may achieve an anti-oxidative state in neutrophils via mechanisms related to “hormesis,” a process in which exposure to sub-threshold stimulations that are damaging at higher doses induces an adaptive beneficial effect. Thus, low concentrations of ROS may activate a repair system, while high concentrations of ROS induce damaging effects (cell death). A single bout of ASE generated large amounts of ROS (∼45% increase) and accelerated neutrophil apoptosis (Figs. 1 and 2). In contrast, acute moderate exercise (AME) only slightly increased neutrophil ROS (∼10%) without altering apoptosis-related parameters (Fig. S4). When subjects performed AME regularly during CME, their neutrophils were repeated exposed to small amount of ROS and might accumulate antioxidants accordingly. As a matter of fact, similar mechanisms have been demonstrated in studying exercise effects on the skeletal muscle. Acute exercise not only generates ROS via activating xanthine oxidase but also transiently induces antioxidant mRNA expression via activating NF-κB in the skeletal muscle [52], [53]. Moreover, the beneficial effects of exercise training in terms of over-expression of antioxidant enzymes are blocked by inhibiting xanthine oxidase [54], [55].

The beneficial effects of CME on neutrophils were rather long-lasting, i.e., most of them were durable after 2 months of DT. Likewise, the CME-improved exercise performance (ASE duration and maximum workload) did not fully reverse after DT for 2 months either. Our preliminary results showed that the citrate synthase activity in neutrophils was elevated by CME and it remained high after DT as well (data not shown). Therefore, CME persistently elevated the aerobic capacity not only in skeletal muscles but more so in neutrophils. In short-lived neutrophils, such long-lasting changes could occur in the bone marrow where their progenitor cells undergo functional differentiation [56]. However, mechanistically it is still unknown how the CME effects persist on neutrophils after detraining. Nevertheless, the CME effects on physiological parameters (body weight, body mass index, resting heart rate) reverted to the pre-training state in just 1 month after ceasing regular exercise, probably due to the unbalanced food intake and energy expenditure during the DT period.

In addition to the opposing effects of CME and ASE on neutrophil apoptosis, CME actually prevented the ASE-evoked neutrophil deficits (Table 2). Since CME significantly improved exercise capacity, neutrophils from subjects with different levels of physical fitness are likely to behave differently as well. Therefore, different physical fitness of subjects and different exercise protocols used in various studies would greatly influence the experimental outcome [23]–[27]. As mentioned earlier, the CME effects were relatively long-lasting. Thus CME may be beneficial to patients suffering acquired neutropenia, especially the disorders due to elevated neutrophil apoptosis [57]–[59]. For example, patients under chemotherapy or with HIV infection could be benefited by taking regular moderate exercise to prolong their shortened lifespan of neutrophils.

This study not only provides insight into mechanisms important for explaining how different exercise paradigms affect neutrophil spontaneous apoptosis but also points out a practical way to improve our innate immunity. Interestingly, our early studies have shown that ASE makes platelet hyperactive in sedentary subjects and CME exerts opposite effects [33], [34]. Taken together, it would be advisable to perform CME but to avoid ASE in general.

## Supporting Information

Figure S1 **Fluorescence staining of neutrophils.** Fluorescence stained neutrophils were allowed to adhere to the glass slide for 20 min before being examined under a microscope. Freshly isolated neutrophils were stained by DCF-DA at rest (A) or after being stimulated by PMA for 10 min (B). Freshly isolated neutrophils were stained by JC-1, which formed green monomers under low ΔΨm (C) and red aggregates under high ΔΨm (D). Neutrophils cultured for 10 h were stained with Ann-V (E) and MitoSOX (F) to show apoptosis and mtROS, respectively.
(TIF)

Figure S2 **Similar effects of initial ASE and H_2_O_2_ exposure on neutrophil ΔΨm.** (A, B): at the beginning of the program, blood specimens were obtained from a sedentary subject both at rest and immediately after ASE. Neutrophils were cultured for 10 h and then analyzed for ΔΨm. (C, D): resting neutrophils were isolated from another sedentary subject. Neutrophils before and after being exposed to 100 µM H_2_O_2_ for 30 min. They were cultured for 10 h and then analyzed for ΔΨm.
(TIF)

Figure S3 **Similar effects of initial ASE and H_2_O_2_ exposure on neutrophil Ann-V binding and ΔΨm.** Neutrophils were double stained by Ann-V and MitoTracker Red to show apoptotic cells (Ann-V^+^ cells %, labeled in the bottom) and depolarized ΔΨm (dimmed MiroTracker Red). (A, B): at the beginning of the program, blood specimens were obtained from a sedentary subject both at rest and immediately after ASE. Neutrophils were cultured for 10 h and then analyzed for Ann-V binding and ΔΨm. (C, D): resting neutrophils were isolated from another sedentary subject. Neutrophils before and after being exposed to 100 µM H_2_O_2_ for 30 min. They were cultured for 10 h and then analyzed for Ann-V binding and ΔΨm.
(TIF)

Figure S4 **Effects of acute moderate exercise (AME) on neutrophil parameters related to ROS and apoptosis.** Five additional sedentary subjects were recruited and fulfilled the same criteria as described in the methods section. They underwent an ASE to determine the maximal workload. Two days after ASE, they performed an AME for 30 min (60% of maximal workload). Neutrophils were isolated from blood drawn at rest and immediately after AME. The ROS-related parameters were analyzed immediately after neutrophil isolation (A, B), whereas the apoptosis-related parameters were determined after 10 h incubation in culture (C–E). Data were analyzed by paired *t*-test. * *p*<0.05, after ASE *vs* resting; n = 5.
(TIF)
