# Supplementary figures and images for: Severe Exercise and Exercise Training Exert Opposite Effects on Human Neutrophil Apoptosis via Altering the Redox Status
Source: PLoS One. 2011 Sep 9;6(9):e24385. doi: 10.1371/journal.pone.0024385 (PMC3170310; doi:10.1371/journal.pone.0024385)

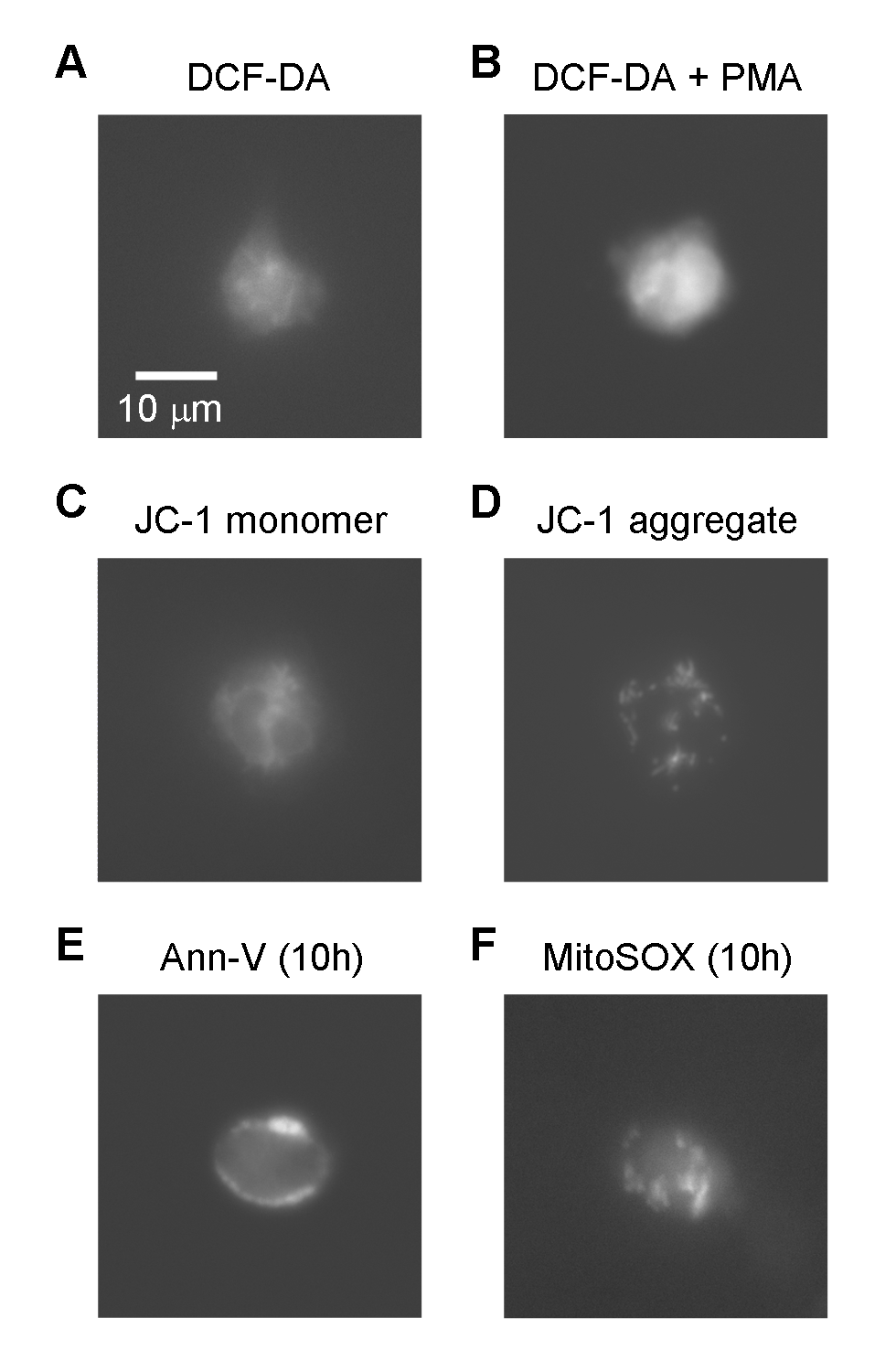

Supplement: Figure S1 — Fluorescence staining of neutrophils. Fluorescence stained neutrophils were allowed to adhere to the glass slide for 20 min before being examined under a microscope. Freshly isolated neutrophils were stained by DCF-DA at rest (A) or after being stimulated by PMA for 10 min (B). Freshly isolated neutrophils were stained by JC-1, which formed green monomers under low ΔΨm (C) and red aggregates under high ΔΨm (D). Neutrophils cultured for 10 h were stained with Ann-V (E) and MitoSOX (F) to show apoptosis and mtROS, respectively. (TIF) [file pone.0024385.s001.tif]

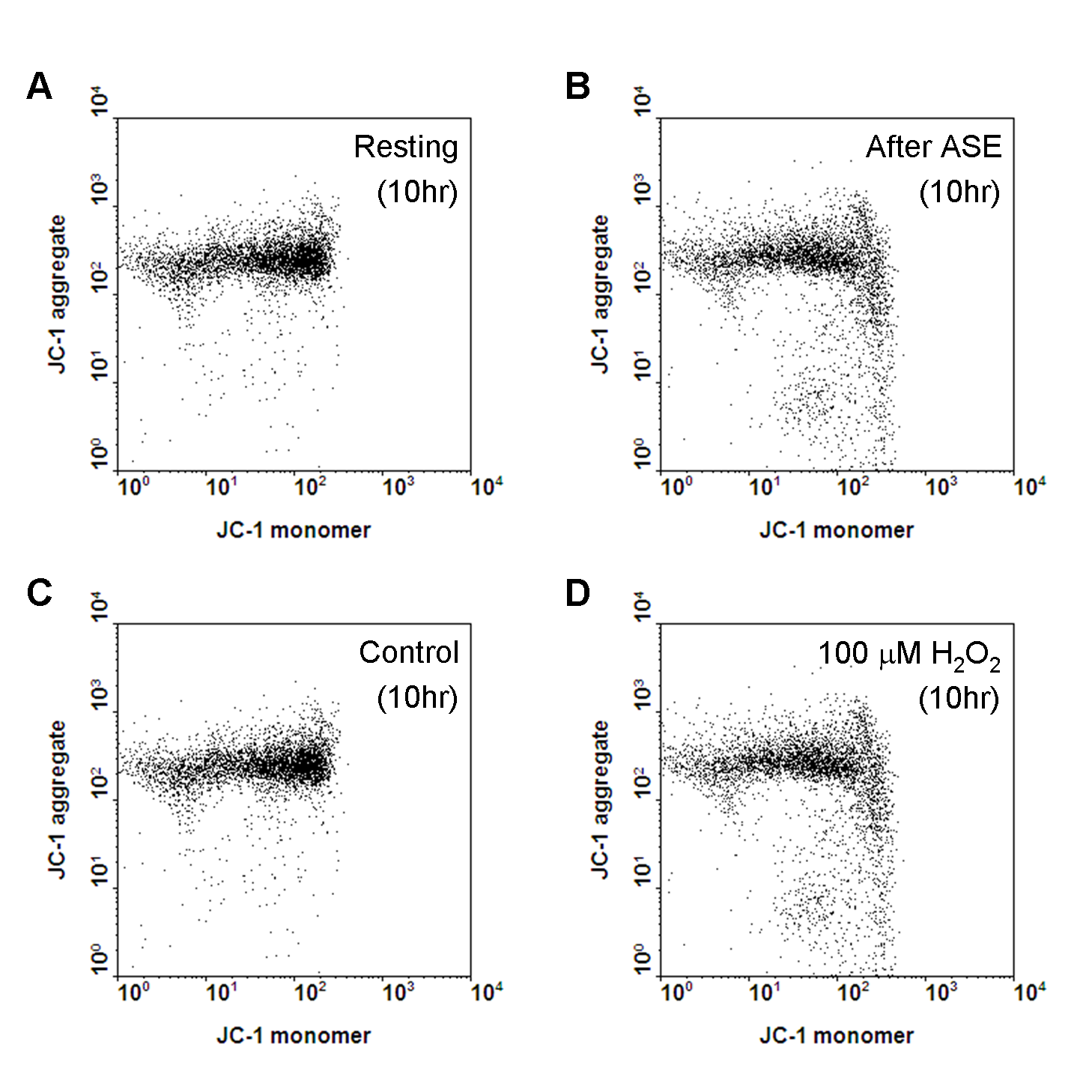

Supplement: Figure S2 — Similar effects of initial ASE and H2O2 exposure on neutrophil ΔΨm. (A, B): at the beginning of the program, blood specimens were obtained from a sedentary subject both at rest and immediately after ASE. Neutrophils were cultured for 10 h and then analyzed for ΔΨm. (C, D): resting neutrophils were isolated from another sedentary subject. Neutrophils before and after being exposed to 100 µM H2O2 for 30 min. They were cultured for 10 h and then analyzed for ΔΨm. (TIF) [file pone.0024385.s002.tif]

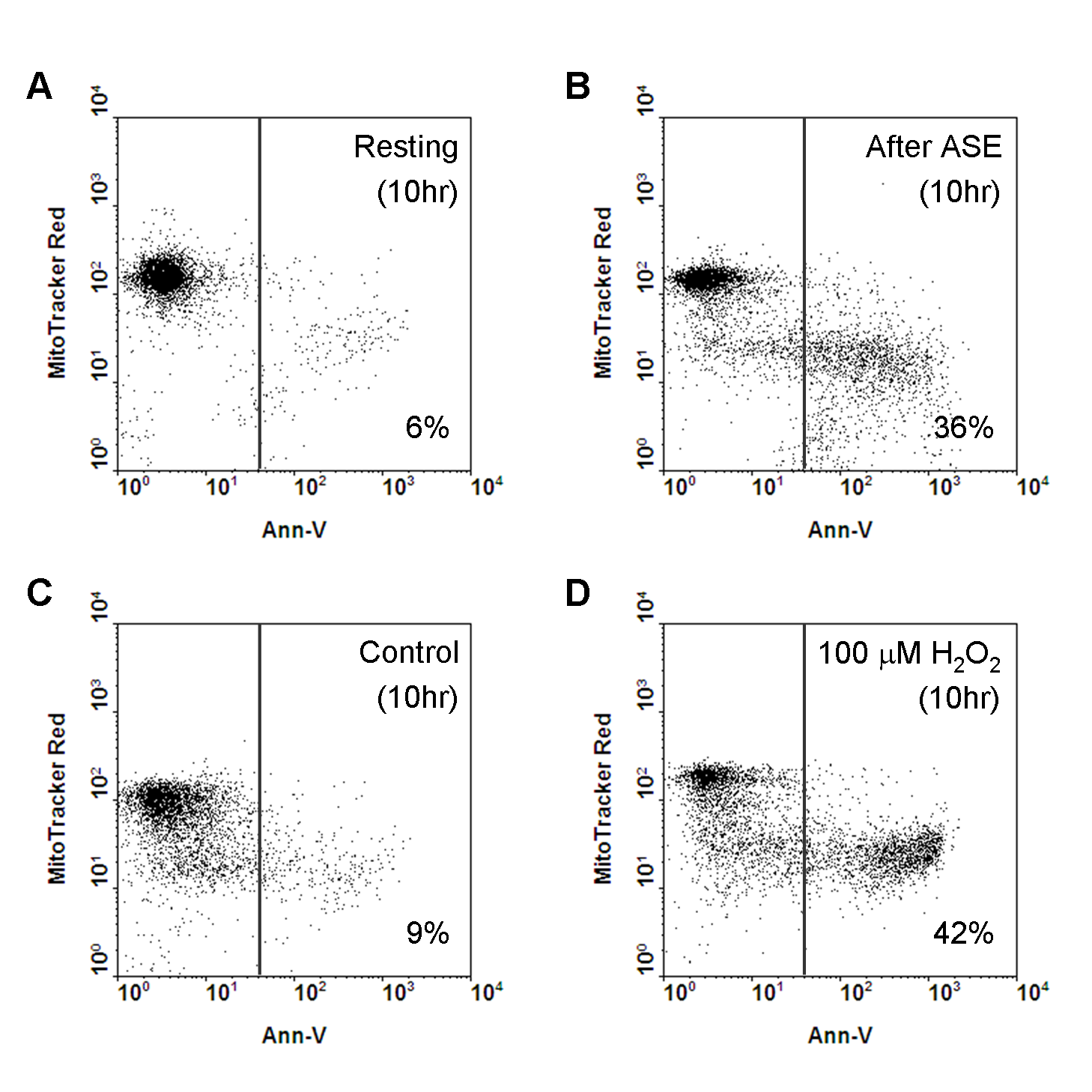

Supplement: Figure S3 — Similar effects of initial ASE and H2O2 exposure on neutrophil Ann-V binding and ΔΨm. Neutrophils were double stained by Ann-V and MitoTracker Red to show apoptotic cells (Ann-V+ cells %, labeled in the bottom) and depolarized ΔΨm (dimmed MiroTracker Red). (A, B): at the beginning of the program, blood specimens were obtained from a sedentary subject both at rest and immediately after ASE. Neutrophils were cultured for 10 h and then analyzed for Ann-V binding and ΔΨm. (C, D): resting neutrophils were isolated from another sedentary subject. Neutrophils before and after being exposed to 100 µM H2O2 for 30 min. They were cultured for 10 h and then analyzed for Ann-V binding and ΔΨm. (TIF) [file pone.0024385.s003.tif]
